# Phase 2 study of irinotecan plus cetuximab rechallenge as third-line treatment in *KRAS* wild-type metastatic colorectal cancer: JACCRO CC-08

**DOI:** 10.1038/s41416-020-01042-w

**Published:** 2020-08-31

**Authors:** Toshiki Masuishi, Akihito Tsuji, Masahito Kotaka, Masato Nakamura, Mitsugu Kochi, Akinori Takagane, Ken Shimada, Tadamichi Denda, Yoshihiko Segawa, Hiroaki Tanioka, Hiroki Hara, Tamotsu Sagawa, Takanori Watanabe, Takao Takahashi, Yuji Negoro, Dai Manaka, Hideto Fujita, Takeshi Suto, Masahiro Takeuchi, Wataru Ichikawa, Masashi Fujii

**Affiliations:** 1 Department of Clinical Oncology, Aichi Cancer Center Hospital, 1-1 Kanokoden, Chikusa-ku, Nagoya, Aichi 464-8681 Japan; 2 Department of Gastroenterology, Tsuchiura Kyodo General Hospital, 1-1, Ootsuno, Tsuchiura, Ibaraki 300-0028 Japan; 3 Department of Medical Oncology, Kagawa University Hospital, 1750-1 Ikenobe, Miki-cho, Kita-gun, Kagawa 761-0793 Japan; 4 Gastrointestinal Cancer Center, Sano Hospital, 2-5-1 Shimizugaoka, Tarumi-ku, Kobe, Hyogo 655-0031 Japan; 5 Aizawa Comprehensive Cancer Center, Aizawa Hospital, 2-5-1 Honjyou, Matsumoto, Nagano 390-8510 Japan; 6 Department of Digestive Surgery, Nihon University School of Medicine, 30-1 Oyaguchikamimachi, Itabashi-ku, Tokyo 173-8610 Japan; 7 Department of Surgery, Hakodate Goryoukaku Hospital, 38-3 Goryoukaku-cho, Hakodate, Hokkaido 040-8611 Japan; 8 Department of Internal Medicine, Showa University Koto Toyosu Hospital, 5-1-38 Toyosu, Koto-ku, Tokyo 135-8577 Japan; 9 Department of Gastroenterology, Chiba Cancer Center, 666-2 Nitona-cho, Chuo-ku, Chiba 350-1298 Japan; 10 Department of Medical Oncology, Saitama Medical University International Medical Center, 1397-1 Yamane, Hidaka, Saitama 350-1298 Japan; 11 Clinical Oncology, Kawasaki Medical School Hospital, 577 Matsushima, Kurashiki, Okayama 701-0192 Japan; 12 Department of Gastroenterology, Saitama Cancer Center, 780 Komuro, ina, Kita-adachi-gun, Saitama 362-0806 Japan; 13 Department of Gastroenterology, Hokkaido Cancer Center, 2-3-54 Kikusuishijyo, Shiroishi-ku, Sapporo, Hokkaido 003-0804 Japan; 14 Department of Surgery, Japanese Red Cross Society Himeji Hospital, 1-12-1 Shimoteno, Himeji, Hyogo 670-8540 Japan; 15 Department of Surgical Oncology, Gifu University, Graduate School of Medicine, 1-1 Yanagido, Gifu, 501-1194 Japan; 16 Department of Gastroenterology, Kochi Health Sciences Center, 2125-1 Ike, Kochi, 781-8555 Japan; 17 Department of Surgery, Kyoto Katsura Hospital, 17 Yamadahirao-cho, Nishikyo-ku, Kyoto 615-8256 Japan; 18 Department of Digestive Surgery, Kanazawa Medical University Hospital, 1-1 Daigaku, Uchinadamachi, Kahoku-gun, Ishikawa 920-0293 Japan; 19 Department of Gastroenterological Surgery, Yamagata Prefectural Central Hospital, 1800 Ooazaaoyagi, Yamagata, 990-2292 Japan; 20 Department of Clinical Medicine (Biostatistics), Kitasato University School of Pharmacy, 5-9-1 Shirokane, Minato-ku, Tokyo 108-8641 Japan; 21 Division of Medical Oncology, Showa University Fujigaoka Hospital, 1-30 Fujigaoka, Aoba-ku, Yokohama, Kanagawa 227-8501 Japan

**Keywords:** Targeted therapies, Colorectal cancer

## Abstract

**Background:**

Regorafenib or trifluridine/tipiracil as third-line treatment have limited efficacy in metastatic colorectal cancer (mCRC).

**Methods:**

This Phase 2 trial evaluated the efficacy and safety of irinotecan plus cetuximab rechallenge as third-line treatment in *KRAS* wild-type mCRC patients who achieved clinical benefit with first-line cetuximab-containing therapy. The primary endpoint was 3-month progression-free survival (PFS) rate. A sample size was calculated; 30 patients with a 3-month PFS rate of 45% deemed promising and 15% unacceptable. Patients with greater and less than the cut-off value of cetuximab-free intervals (CFIs) were classified into the long and short CFI groups, respectively, in subgroup analyses.

**Results:**

Among 34 eligible patients who received treatment at least once, 3-month PFS rate was 44.1% (95% confidence interval, 27.4–60.8%). The median PFS and overall survival (OS) were 2.4 and 8.2 months, respectively. The response and disease control rates were 2.9 and 55.9%, respectively. PFS and OS were significantly longer in the long- than in the short CFI group.

**Conclusions:**

Irinotecan plus cetuximab rechallenge as third-line treatment for *KRAS* wild-type mCRC was safe and had promising activity, especially in those with a long CFI, warranting further investigation in a Phase 3 randomised trial.

**Clinical trial registration:**

UMIN000010638

## Background

5-fluorouracil/leucovorin plus oxaliplatin or irinotecan (FOLFOX or FOLFIRI) in combination with an anti-epidermal growth factor receptor (EGFR) antibody (cetuximab or panitumumab) is a standard first- or second-line therapy in patients with *RAS* wild-type metastatic colorectal cancer (mCRC).^1–3^ In addition, anti-EGFR antibodies with or without irinotecan are used as standard third-line treatment in patients who have not been previously treated with anti-EGFR antibodies.^4–6^ Patients who are refractory to these drugs can receive regorafenib or trifluridine/tipiracil (FTD/TPI) as a third-line or later treatment option. Although regorafenib and FTD/TPI were shown to achieve a significantly longer overall survival (OS) than placebo in the CORRECT and RECOURSE trials, these drugs have limited efficacy, with a response rate (RR) ranging from 1 to 1.6%.^7,8^ The development of new drugs and strategies is necessary to improve outcomes in patients receiving third-line treatment.

Santini et al. reported that cetuximab rechallenge had promising efficacy in patients who achieved clinical benefit in response to first-line cetuximab plus chemotherapy. The RR of FOLFIRI (or irinotecan) plus cetuximab rechallenge was 54%, which was much higher than that of regorafenib or FTD/TPI. However, the study by Santini et al. was a single-arm Phase 2 study, and no study had reproduced the efficacy of cetuximab rechallenge at that time.^9^ Therefore, we conducted a Phase 2 study of irinotecan plus cetuximab rechallenge as third-line treatment in patients with *KRAS* exon 2 wild-type mCRC (Clinical trial information: UMIN000010638).

## Methods

### Study design and treatment schedule

This was a Phase 2, multicentre, open-label, non-randomised trial to evaluate the efficacy and safety of irinotecan plus cetuximab rechallenge as third-line treatment in patients with *KRAS* exon 2 wild-type mCRC. Patients received 150 mg/m^2^ irinotecan intravenously every 2 weeks. Cetuximab was administered as a 2-h intravenous infusion at a loading dose of 400 mg/m^2^, followed by weekly 1-h infusions of 250 mg/m^2^. Irinotecan was allowed to be discontinued after day 15 based on the investigator’s decision. All patients received an H1-histamine antagonist and dexamethasone before cetuximab and 5-hydroxytryptamine-3 receptor antagonist before irinotecan.

The protocol for the present study was reviewed and approved by the Institutional Review Boards of all participating institutions.

### Eligibility criteria

Inclusion criteria were (1) histologically confirmed unresectable colorectal adenocarcinoma, (2) *KRAS* exon 2 wild-type, (3) achievement of complete response, partial response or stable disease for at least 6 months with first-line cetuximab plus doublet chemotherapy, (4) refractoriness or intolerance to two regimens, including oxaliplatin and irinotecan, (5) Eastern Cooperative Oncology Group performance status (ECOG PS) of 0–2, (6) measurable lesion according to the Response Evaluation Criteria in Solid Tumors (RECIST) version 1.1, (7) adequate bone marrow, hepatic and renal function (white blood cell count, ≥3000/μL and ≤12,000/μL; neutrophil count, ≥1500/μL; haemoglobin, ≥9.0 g/dL; platelet count, ≥100,000/μL; total bilirubin, ≤2.0 mg/dL; aspartate and alanine aminotransferase, ≤100 IU/L or ≤300 IU/L in patients with liver metastases; creatinine, ≤1.5 mg/dL), (8) age > 20 years, (9) life expectancy of at least 3 months and (10) written informed consent. Exclusion criteria were (1) history of malignant tumours within 5 years before the study treatment initiation, (2) symptomatic brain metastases, (3) severe complications (infection, lung and heart disease and liver and renal dysfunction), (4) massive pleural effusion, ascites and pericardial effusion and (5) other serious medically important abnormalities.

### Toxicity and dose modifications

Toxicity was assessed according to the National Cancer Institute Common Toxicity Criteria (version 4.0). In the presence of ≥grade 3 skin toxicity, cetuximab dose interruption was required until regression to ≤grade 2. If grade ≥3 skin toxicity occurred two times, the dose of cetuximab was reduced to 200 mg/m^2^, and if grade ≥3 skin toxicity occurred once more, the dose of cetuximab was reduced to 150 mg/m^2^. In the presence of ≥grade 2 diarrhoea and ≥grade 3 haematological toxicity, irinotecan dose interruption was required until regression to ≤grade 1 and ≤grade 2, respectively. In the presence of ≥grade 3 haematological toxicity and thrombocytopenia and grade 4 neutropenia, irinotecan dose was sequentially reduced to 120 and 100 mg/m^2^. Supportive therapy was administered when necessary. The protocol treatment was repeated until disease progression, unacceptable toxicity, death or withdrawal of consent.

### Outcome measures and statistical analysis

All patients underwent computed tomography (CT) at chemotherapy initiation and every 8 weeks to evaluate tumour response according to RECIST version 1.1. The primary endpoint was 3-month progression-free survival (PFS). A sample size was calculated; 30 patients with a 3-month PFS rate of 45% deemed promising and 15% unacceptable (one-sided α, 0.05; β, 0.2). The secondary endpoints were RR, disease control rate (DCR), PFS, OS, time to treatment failure (TTF) and safety. RR was defined as the proportion of patients with complete or partial response. DCR was defined as the proportion of patients with complete, partial response or stable disease. Complete, partial response and stable disease were without confirmation of response. PFS was defined from the date of enrolment to the first observation of disease progression or death from any cause. OS was defined from the date of enrolment to death from any cause. PFS and OS were estimated using the Kaplan–Meier method. Deepness of response (DpR) defined as the rate of tumour shrinkage from baseline CT was evaluated as an exploratory analysis.

Post hoc subgroup analysis according to cetuximab-free interval (CFI) was conducted to explore predictive factors for irinotecan plus cetuximab rechallenge. CFI was defined as the interval from the date of the last cetuximab dose in first-line treatment to the date of the first cetuximab dose in the present study. Receiver-operating characteristic (ROC) analysis using a 3-month PFS rate as the second variable was performed to determine an optimal cut-off value of CFI. Patients with greater and less than the optimal cut-off value for CFI were classified into long and short CFI groups, respectively. In subgroup analyses, DCR, rate of DpR > 0%, PFS and OS were compared between the two CFI groups. Several cut-off values of CFI based on a previous report^10^ were used as it was unclear whether the cut-off value of CFI in the present study was appropriate. All analyses were conducted using SAS version 9.2 (SAS Institute, Cary, NC, USA) and R version 3.4.1 (The R Foundation for Statistical Computing, Vienna, Austria).

## Results

### Patient characteristics

The study included 36 patients enrolled between May 2013 and October 2015. One patient could not receive protocol treatment due to rapid disease progression after enrolment in the study. The safety population included patients who received the protocol treatment at least once. Thirty-four of the 35 patients received the protocol treatment. One patient proved to be ineligible after initiation of protocol treatment because no blood evaluation had been performed for this patient before study enrolment. The full analysis set comprised patients meeting the eligibility criteria who received the protocol treatment at least once, and included 34 patients after the exclusion of one ineligible patient.

The study cohort characteristics are summarised in Table 1. Briefly, the median age was 64.5 (range, 41–80) years, 11 patients (32%) were female, 33 patients (97%) had an ECOG PS of 0–1, primary colorectal tumours were not resected in 9 patients (27%) at the time of enrolment in this study, 30 patients (88%) had left-sided tumours (rectum, sigmoid or descending colon), 29 patients (85%) had liver metastases, 8 patients (24%) had peritoneal metastases and 25 patients (77%) had two or more metastatic sites. With first-line cetuximab-containing treatments, 29 patients (85%) had partial response and 5 patients (15%) had stable disease for 6 months or more. In addition, 15 patients (44%) received irinotecan-based regimens as first-line treatment; all 15 patients received oxaliplatin-based regimens as second-line treatment. Among 34 patients, 33 of the 34 patients (97%) received bevacizumab as second-line treatment.

**Table 1:** Patient characteristics.

Characteristics		*N* = 34 (%)	
Age	Median (years) (range)	64.5	(41–80)
Sex	Male	23	(68)
	Female	11	(32)
ECOG PS	0	20	(59)
	1	13	(38)
	2	1	(3)
Site of primary tumour	Right-sided colon^a^	4	(12)
	Left-sided colorectum^b^	30	(88)
Prior resection of the primary tumour	Yes	25	(74)
	No	9	(26)
Metastatic sites	Liver	29	(85)
	Lung	14	(41)
	Peritoneum	8	(24)
No. of metastatic sites	1	9	(26)
	2 or 3	19	(56)
	≥4	6	(18)
First-line Tx	Oxaliplatin-based	19	(56)
	Irinotecan-based	15	(44)
Best overall response in first-line Tx	CR	0	0
	PR	29	(85)
	SD	5	(15)
Second-line Tx	Oxaliplatin-based	15	(44)
	Irinotecan-based	19	(56)
	Bevacizumab	33	(97)
Best response in second-line Tx	PR	5	(15)
	SD	27	(79)
	PD	2	(6)

*ECOG* Eastern Cooperative Oncology Group, *PS* performance status, *No.* number, *Tx* treatment.

^a^Caecum, ascending and transverse colon.

^b^Descending and sigmoid colon and rectum.

### Treatment exposure

The median number of doses was 8 (range, 1–37) for cetuximab and 4 (range, 1–13) for irinotecan, whereas the median relative dose intensity was 80.3% (60.2 mg/m^2^/week; range, 22.7–80.6 mg/m^2^/week) for irinotecan and 90.1% (225.3 mg/m^2^/week; range, 66.0–280.5 mg/m^2^/week) for cetuximab. Irinotecan and cetuximab dose modifications were required in 8 (23.5%) and 5 (14.7%) patients, respectively, due to adverse events. Irinotecan and cetuximab dose interruptions were required in 18 (52.9%) and 17 (50.0%) patients, respectively. All patients had discontinued irinotecan plus cetuximab at the time of analysis. The median follow-up duration was 13.2 (range, 1.1–29.2) months, and the median TTF was 2.2 months (95% confidence interval [CI], 1.8–3.2 months). The reasons for discontinuation included disease progression in 32 patients (94%), grade 3 skin toxicities that were not resolved within 35 days from the last dose of cetuximab in one patient (3%) and grade 2 anorexia in one patient (3%). There were no treatment-related deaths during the study period.

### Efficacy

**Fig. 1:**
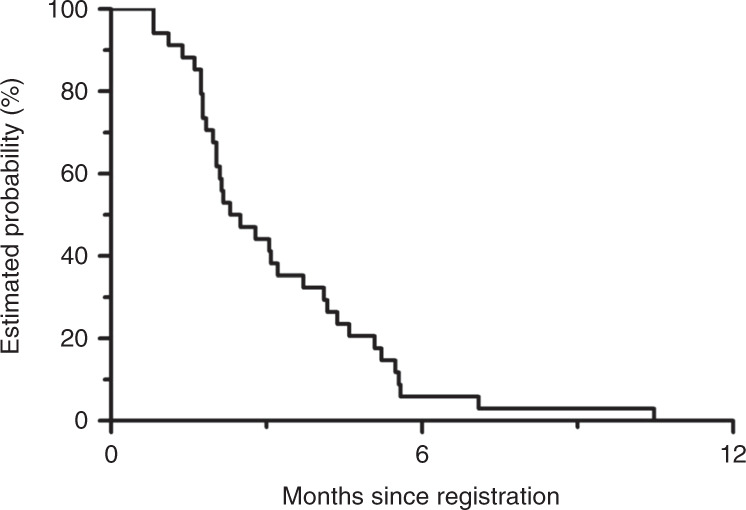
Progression-free survival. The median progression-free survival and 3-month progression-free survival rate are 2.4 months (95% CI, 2.0–3.7 months) and 44.1% (95% CI, 27.4–60.8%). CI confidence interval.

**Fig. 2:**
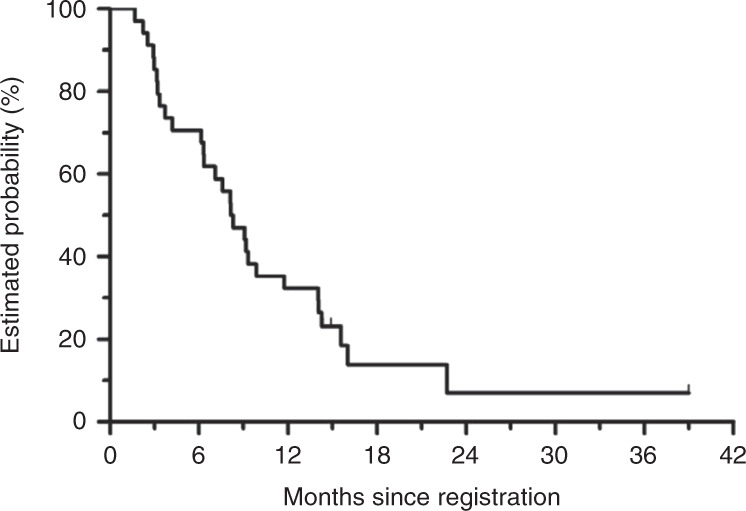
Overall survival. The median overall survival is 8.2 months (95% CI, 6.1–11.7 months). CI confidence interval.

Among 34 patients in the full analysis set, 3-month PFS rate was 44.1% (95% CI, 27.4–60.8%) (Fig. 1). The median PFS and OS were 2.4 months (95% CI, 2.0–3.7 months) and 8.2 months (95% CI, 6.1–11.7 months), respectively (Figs. 1, 2). One patient achieved partial response with an RR of 2.9% (95% CI, 0.07–15.3%), and 18 patients achieved stable disease with a DCR of 55.9% (95% CI, 37.9–72.8%). The median DpR was −6.7% (range, −66.0 to 31.7%), and 9 patients (27%) had a DpR of > 0%.

### Subgroup analysis according to CFI

The median CFI was 330 (range, 56–1224) days. The optimal CFI cut-off value of 372 days was determined by ROC analysis (area under the curve = 0.81).

**Fig. 3:**
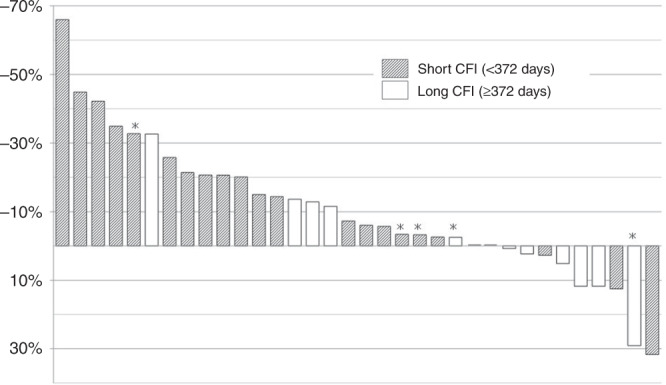
Deepness of response by cetuximab-free interval. The rate of deepness of response >0% is higher in the long- than in the short CFI group (55% vs. 13%, respectively, *p* = 0.003). The CFIs of three patients with a rate of DpR of >0% in the short CFI group were 338, 181 and 176 days, respectively, from the right of patients. *Patients who discontinued first-line cetuximab-containing treatment due to reasons other than progressive disease. CFI cetuximab-free interval, DpR deepness of response.

**Fig. 4:**
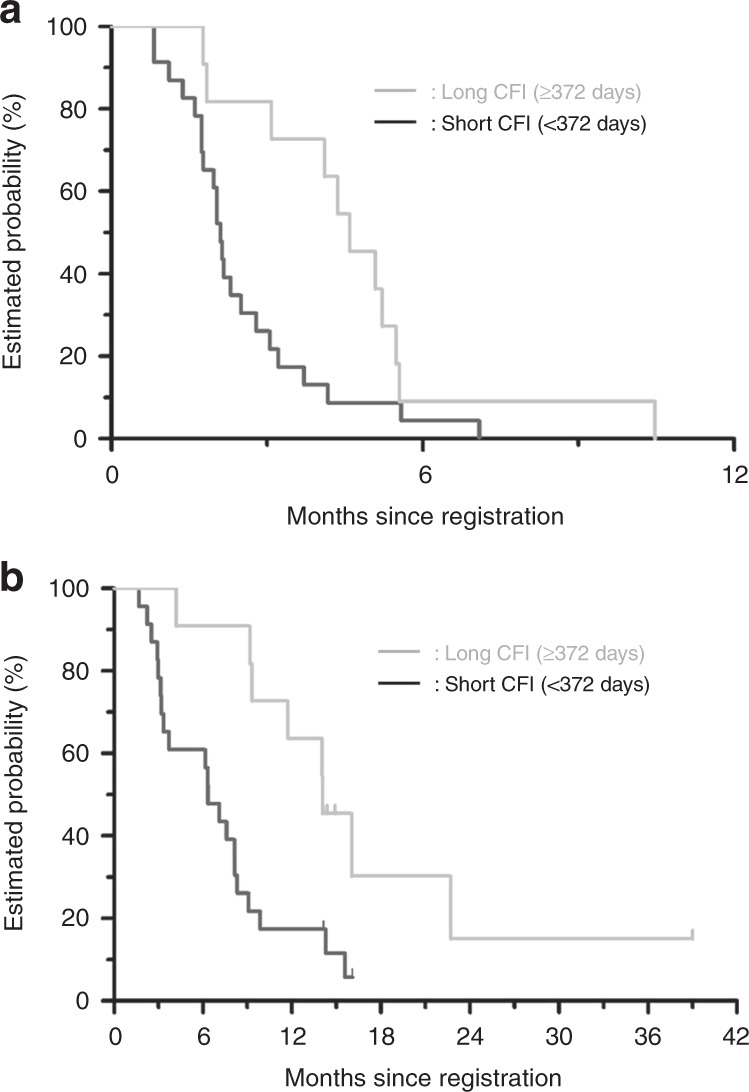
Progression-free survival (a) and overall survival (b) by cetuximab-free interval. **a** The median progression-free survival is 4.6 months in the long CFI group and 2.1 months in the short CFI group (hazard ratio, 0.40; 95% CI, 0.18–0.86; *p* = 0.020). **b** The median overall survival is 14.6 months in the long CFI group and 6.3 months in the short CFI group (hazard ratio, 0.31; 95% CI, 0.13–0.74; *p* = 0.008). CFI cetuximab-free interval, CI confidence interval.

Among 34 patients, 11 and 23 patients were classified into the long and short CFI groups. The DCRs were 82% and 44% for the long and short CFI groups, respectively (*p* = 0.064). The rate of DpR of >0% was higher in the long- than in the short CFI group (55% vs. 13%, respectively, *p* = 0.033) (Fig. 3). The CFIs of three patients with a rate of DpR of >0% in the short CFI group were 338, 181 and 176 days, respectively, from the right of patients in Fig. 3. The 3-month PFS rates were 81.8% and 26.1% in the long and short CFI groups, respectively. The median PFS was 4.6 months in the long CFI group and 2.1 months in the short CFI group (hazard ratio, 0.40; 95% CI, 0.18–0.86; *p* = 0.020) (Fig. 4a). The median OSs were 14.1 and 6.3 months in the long and short CFI groups, respectively (hazard ratio, 0.31; 95% CI, 0.13–0.74, *p* = 0.008) (Fig. 4b).

Using several cut-off values of CFI, including 4.4, 6 and 8.8 months, the PFS and OS were longer in the long- than in the short CFI group (Supplementary Figs. 1, 2 and Supplementary Table 1).

### Adverse events

Among 35 patients in the safety population, common grade 3–4 adverse events included neutropenia (*n* = 10, 29%), anaemia (*n* = 2, 6%), elevated aspartate aminotransferase (*n* = 2, 6%), anorexia (*n* = 2, 6%) and fatigue (*n* = 2, 6%). There were no grade 3–4 skin toxicities. Common grade 1–2 skin toxicities were skin rash (*n* = 28, 80%), dry skin (*n* = 20, 57%) and paronychia (*n* = 16, 46%) (Table 2).

**Table 2:** Adverse events.

	*N* = 34			
	All grades (%)		≥Grade 3 (%)	
Neutropenia	13	(37)	10	(29)
Anaemia	19	(54)	2	(6)
Thrombocytopenia	5	(14)	1	(3)
Febrile neutropenia	1	(3)	1	(3)
Anorexia	23	(66)	2	(6)
Nausea	12	(34)	0	0
Diarrhoea	8	(23)	0	0
Mucositis	16	(46)	0	0
Fatigue	15	(43)	2	(6)
Alopecia	16	(46)	–	–
Skin rash	28	(80)	0	0
Dry skin	20	(57)	0	0
Paronychia	16	(46)	0	0
Infusion-related reaction	1	(3)	0	0
Hypomagnesaemia	18	(51)	0	0
Increased AST	21	(60)	2	(6)
Increased ALT	18	(51)	1	(3)

## Discussion

In the present Phase 2 study, we demonstrated that irinotecan plus cetuximab rechallenge as third-line treatment was safe and had promising activity in patients with *KRAS* wild-type mCRC who exhibited clinical response to cetuximab plus cytotoxic agents as first-line chemotherapy, warranting further investigation in a Phase 3 randomised trial. Specifically, patients with long CFIs might be good candidates for cetuximab rechallenge compared to those with short CFIs. To our knowledge, the present study is the first to show long CFI as a predictive marker for the efficacy of cetuximab rechallenge.

The current results showing the efficacy of cetuximab rechallenge are different than those reported by Santini et al. who observed an RR of 54% with a median PFS of 6.6 months.^9^ Neither the study by Santini et al. nor the present study defined the inclusion criteria for the period between the date of the last cetuximab dose and the date of disease progression. In the present study, we collected the data at the end of enrolment to determine the causes for discontinuation of cetuximab as first-line treatment. In our cohort, the discontinuation was due to reasons other than progressive disease, such as adverse events in 5 patients (15%), indicating that these patients might have sensitivity to cetuximab. Although the study by Santini et al. did not report the causes for cetuximab discontinuation as first-line treatment, it remains possible that the rate of patients who discontinued cetuximab as first-line treatment due to reasons other than progressive disease was higher in their study compared with the present study. In the CRICKET trial, a Phase 2 study of cetuximab plus irinotecan rechallenge, the RR, DCR, median PFS and OS were 21%, 54%, 3.4 months and 9.8 months, respectively. Except for the RR, the efficacy outcomes were comparable between the CRICKET trial and the present study. One cause for the higher RR observed in the CRICKET trial might be due to the inclusion of patients with *RAS* and *BRAF* V600E wild-type mCRC, which was different from the present study.^11^ Therefore, a future Phase 3 study should enrol patients with *RAS/BRAF* wild-type mCRC. In addition, only left-sided mCRC patients should be eligible because previous studies have shown that right-sided mCRC patients had poor responses to anti-EGFR antibodies. In a Phase 2 study of panitumumab plus irinotecan rechallenge for patients with *KRAS* wild-type mCRC, the JACCRO CC-09, RR, DCR, median PFS and OS were 8%, 50%, 3.1 months and 8.9 months, respectively,^12^ suggesting that the efficacy outcomes of cetuximab rechallenge might be similar to those of panitumumab rechallenge.

During treatment with anti-EGFR antibodies, clonal evolution occurs, and tumour cells acquire gene alterations associated with resistance to anti-EGFR antibodies, such as *KRAS*, *NRAS*, *EGFR*, *MET* and *ERBB2*. In fact, in the CRICKET trial, 5 of the 6 patients who achieved PR did not have *RAS* mutations based on circulating tumour DNA (ctDNA) analysis just before the cetuximab rechallenge.^11^ These gene alterations associated with acquired resistance to anti-EGFR antibodies diminished and eventually disappeared with the lapse of time.^10,13^ This phenomenon provides further support to the superior efficacy of cetuximab rechallenge in patients with long CFIs compared to those with short CFIs observed in the present study. Therefore, CFI may be a surrogate marker for acquired gene alteration status. Although we did not conduct ctDNA analysis just before the cetuximab rechallenge to assess acquired resistance to anti-EGFR antibodies, gene alterations associated with acquired resistance to anti-EGFR antibodies might have disappeared in patients with long CFIs, and might have remained in patients with short CFIs. Therefore, these study results provide valuable information for clinical practice, especially in countries where ctDNA analysis has not yet been approved.

The frequency of skin toxicities associated with cetuximab was lower in the present study compared with the study by Santini et al., and the EPIC study in which patients received cetuximab for the first time.^9,14^ Although the underlying mechanism might be the shorter treatment duration in the present study compared with the other studies, this outcome suggests that cetuximab rechallenge might not increase the rate of cetuximab-associated adverse events.

The present study has several limitations. First, patients with *KRAS* exon 2 wild-type CRC were eligible. However, a few tumours with *RAS* or *BRAF* V600E mutations may have been included. Because only patients who achieved clinical benefit (complete, partial response or stable disease for ≥6 months) with first-line cetuximab-containing therapy were enrolled, we believe that most of the patients with *RAS* or *BRAF* V600E mutations were excluded from the present study. Second, the speed of tumour growth as natural history and efficacies of first-line cetuximab-containing treatment or doublet with or without bevacizumab in second-line treatment may affect CFI. However, the higher rate of DpR of > 0% in the long CFI group might indicate a treatment effect of cetuximab rechallenge rather than natural history. In addition, PFS of first-line doublet plus cetuximab and tumour shrinkage of second-line doublet with or without bevacizumab were not associated with the outcome of cetuximab rechallenge (data not shown). Third, it remains unclear whether the cut-off value of CFI defined by ROC analysis in this study was appropriate. Especially, because even though one responder had a short CFI according to the CFI cut-off value determined by ROC analysis, the patient’s CFI (338 days) was higher than the median CFI. Therefore, further validation studies are needed to confirm the appropriate value of CFI as a predictive marker for the efficacy of anti-EGFR antibody rechallenge.

## Conclusion

This Phase 2 study showed that irinotecan plus cetuximab rechallenge as third-line treatment was safe and has promising activity in patients with *KRAS* wild-type mCRC who had a clinical response to cetuximab plus cytotoxic agents as first-line chemotherapy, warranting further investigation in a Phase 3 randomised trial. Specifically, patients with long CFIs might be good candidates for cetuximab rechallenge, whereas patients with short CFIs might not benefit from the cetuximab rechallenge.

## Supplementary information

Supplementary figure legends

Supplementary Figure 1. Progression-free survival using various cut-off values for cetuximab-free interval

Supplementary Figure 2. Overall survival using various cut-off values for cetuximab-free interval

Supplementary Table 1. Median progression-free survival and overall survival with hazard ratios using various cut-off values for cetuximab-free interval

## Data Availability

The datasets used and/or analysed during the current study are available from the corresponding author on reasonable request.
