# Supplementary material for: Phase 2 study of irinotecan plus cetuximab rechallenge as third-line treatment in KRAS wild-type metastatic colorectal cancer: JACCRO CC-08
Source: Br J Cancer. 2020 Aug 31;123(10):1490–5. doi: 10.1038/s41416-020-01042-w (PMC7652864; doi:10.1038/s41416-020-01042-w)
Supplement: Supplementary file 1 — Supplementary figure legends [file 41416_2020_1042_MOESM1_ESM.docx]

**Supplementary Figure 1. Progression-free survival using various cutoff values for cetuximab-free interval**. Using various cutoff values for CFI, including 4.4, 6, and 8.8, the progression-free survival is longer in the long CFI group than in the short CFI group.

CFI, cetuximab-free interval

**Supplementary Figure 2. Overall survival using various cutoff values for cetuximab-free interval.** Using various cutoff values for CFI including 4.4, 6, and 8.8 months, the overall survival is longer in the long CFI group than in the short CFI group.

CFI, cetuximab-free interval
