# Supplementary figures and images for: Phase 2 study of irinotecan plus cetuximab rechallenge as third-line treatment in KRAS wild-type metastatic colorectal cancer: JACCRO CC-08
Source: Br J Cancer. 2020 Aug 31;123(10):1490–5. doi: 10.1038/s41416-020-01042-w (PMC7652864; doi:10.1038/s41416-020-01042-w)

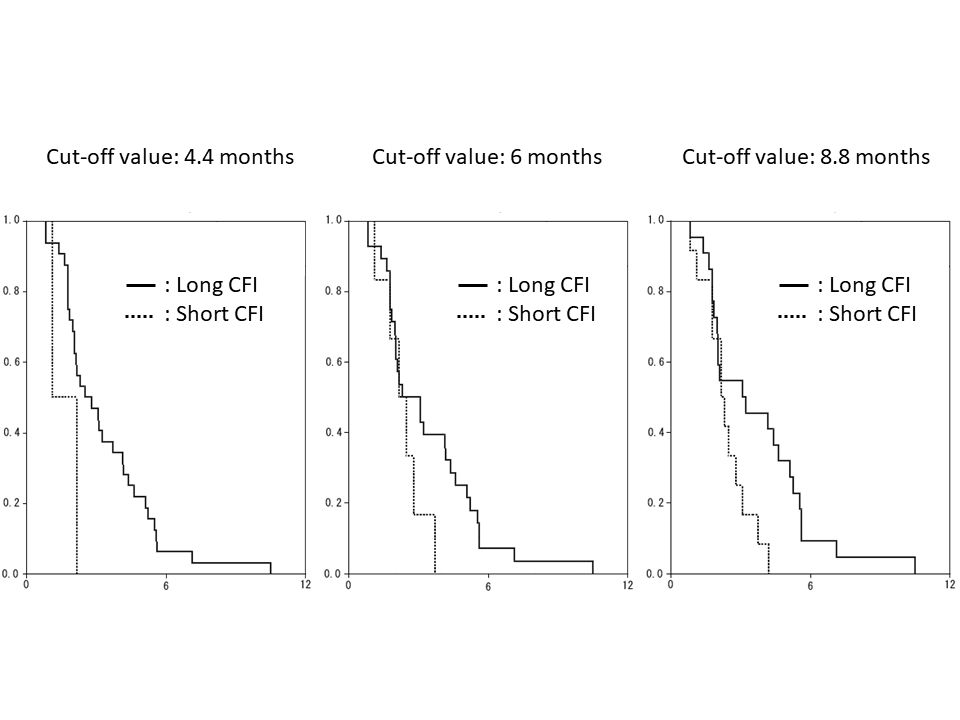

Supplement: Supplementary file 2 — Supplementary Figure 1. Progression-free survival using various cut-off values for cetuximab-free interval [file 41416_2020_1042_MOESM2_ESM.tif]

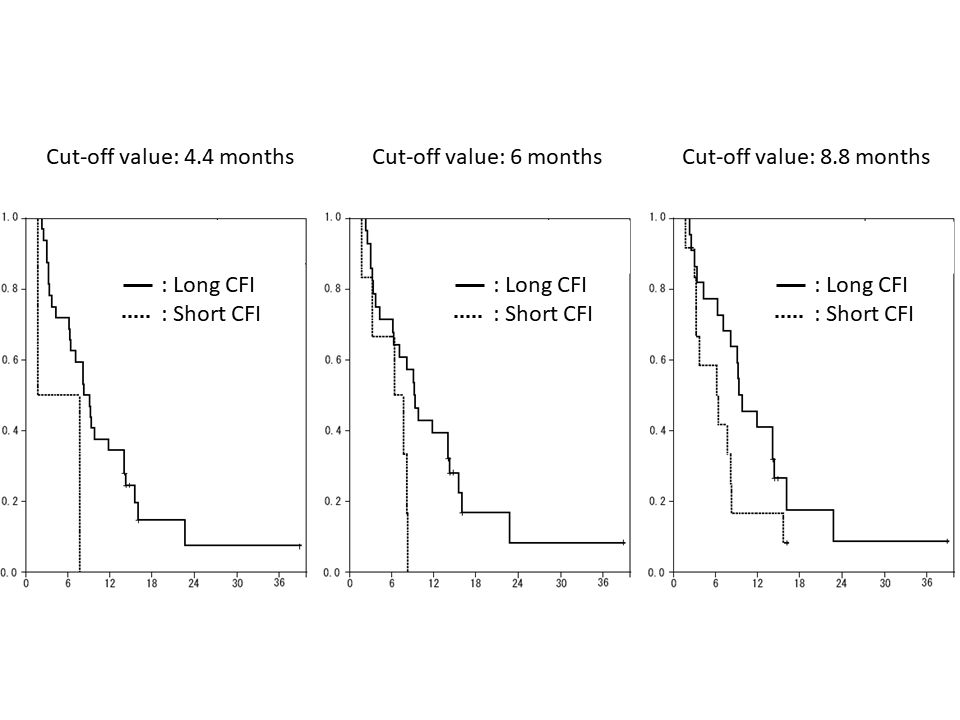

Supplement: Supplementary file 3 — Supplementary Figure 2. Overall survival using various cut-off values for cetuximab-free interval [file 41416_2020_1042_MOESM3_ESM.tif]
